# Patient Safety in a Box: Implementation and Evaluation of the Emergency Box in Geriatric and Parkinson Patients

**DOI:** 10.3390/jcm10235618

**Published:** 2021-11-29

**Authors:** Lea Krey, Pia Lange, Anh Thu Tran, Stephan Greten, Günter U. Höglinger, Florian Wegner, Olaf Krause, Martin Klietz

**Affiliations:** 1 Department of Neurology, Hannover Medical School, Carl-Neuberg-Straße 1, 30625 Hannover, Germany; Tran.AnhThu@mh-hannover.de (A.T.T.); Greten.Stephan@mh-hannover.de (S.G.); Hoeglinger.Guenter@mh-hannover.de (G.U.H.); Wegner.Florian@mh-hannover.de (F.W.); Klietz.Martin@mh-hannover.de (M.K.); 2 Department of General Medicine, Hannover Medical School, Carl-Neuberg-Straße 1, 30625 Hannover, Germany; Krause.Olaf@mh-hannover.de

**Keywords:** emergency box, patient safety, geriatric patients, emergency care, polypharmacy

## Abstract

In an industrial society, the proportion of geriatric people increases with rising age. These people are likely to use polypharmacy and experience medical emergencies. However, their emergency care can be complicated by unclear comorbidities and medication. The aim of this prospective interventional study was to assess the demand for a drug safety tool in clinical practice and to analyze whether the emergency box can improve acute care in a geriatric cohort. Therefore, emergency room (ER) doctors in a German tertiary hospital recorded the number of geriatric patients lacking medical information and its impact on diagnostics/treatment. Furthermore, the emergency box was distributed to patients on the neurological ward and their current drug safety concepts were assessed. After 6 months, we evaluated in a follow-up whether the tool was helpful in emergency cases. Our study revealed that 27.4% (*n* = 28) of the patients came to the ER without their medical information, which caused a relevant delay or possible severe complications in 11.8% (*n* = 12). The emergency box was perceived as easily manageable and 87.9% (*n* = 109) of the participants wanted to keep it after the study. Subjectively, participants benefitted in emergencies. In conclusion, the emergency box is a cheap tool that is easy to use. It can save valuable time in emergencies and increases the safety of geriatric patients.

## 1. Introduction

Due to aging of the population, there is a relevant number of geriatric patients with a complicated medical history and medication scheme [1,2]. These patients are likely to suffer from acute symptoms requiring admittance to the emergency room (ER) [2,3]. The same applies for Parkinson’s disease (PD) patients, as this patient group is very vulnerable and tends to develop treatment complications or acute emergencies [4,5].

We hypothesized that it is a common problem for physicians of geriatric patients to gain access to their medical data upon emergency admittance. The availability of this information can be crucial to decide which diagnostic steps or treatments are necessary, e.g., for thrombolysis therapy in strokes. The lack of patients’ medical data can lead to relevant delays or inaccuracies in acute care or medical treatment.

There have been attempts to establish digital data management systems to overcome this problem all over the world, but only few countries have successfully implemented comprehensive digital solutions so far. Therefore, the Lions Club in Ireland has originally developed the “message in a bottle” concept [6]. It consists of a box capturing important medical information (diagnoses, medication, and allergies). The device is supposed to be stored in the fridge, as this is easy to find in emergencies. The box comes with stickers that are applied to the outside and inside of the front door and the fridge door to alert the rescue services of its existence. The box is supposed to be brought to the ER along with the patient by the rescue services, so all relevant medical information is available immediately. A systematic study proving its acceptance and usefulness in a cohort of geriatric patients has not been performed yet.

The concept has been adapted for Germany, where the emergency box is called “Notfalldose”.

The aim of this prospective interventional study was to assess the demand for a drug safety tool in clinical practice and to analyze whether the emergency box can improve emergency care in a geriatric cohort. 

## 2. Materials and Methods

### 2.1. Ethic Approval and Patient Recruitment

The study was conducted according to the guidelines of the Declaration of Helsinki, and approved by the Ethics Committee of Hannover Medical School (No. 8788_BO_K_2019). All participants gave written and informed consent prior to inclusion. Inclusion criteria for all participants in the study were defined as an age of ≥70 years, multimorbidity (≥3 pre-existing chronic diagnoses), and polypharmacy (≥5 long-term drugs, including over-the-counter (OTC) drugs, as OTC drugs are often used in geriatric patients [7]). This choice of participants is in line with Sieber’s definition of a geriatric patient according to the German Geriatric Society [8]. Because patients from institutional care usually have standard medication plans and charts with relevant medical information these patients were excluded from the study. 

### 2.2. Assessment

**Figure 1:**
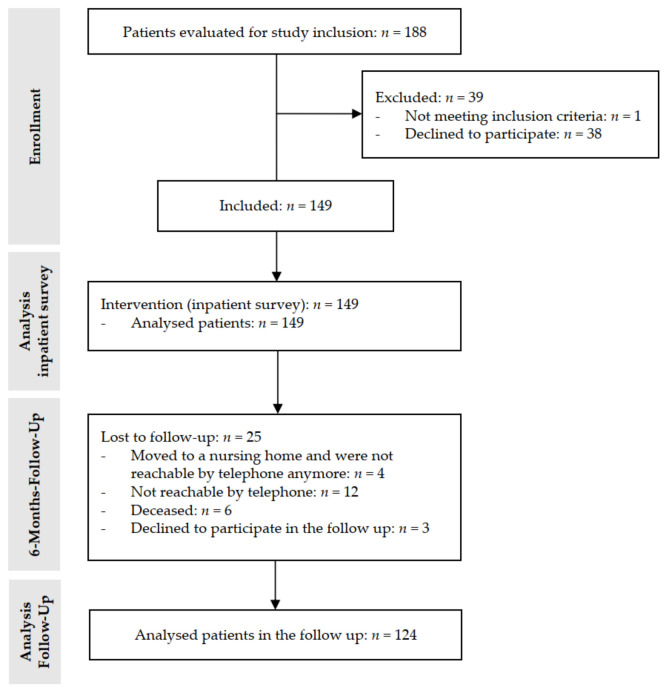
CONSORT flow diagram of the study.

An overview of the study inclusion process can be found in Figure 1.

#### 2.2.1. ER Survey (*n* = 102)

The demand for a medical data management system like the emergency box was assessed by neurological doctors in the ER via anonymous questionnaires (7 questions and demographic data survey) for patients matching the above-mentioned inclusion criteria. We investigated how many geriatric patients came to the ER without the relevant medical information and how many brought a medication scheme or were able to recall their medication from memory correctly. The point of time of the ER visit was recorded (within working hours/at night/on weekends/holidays). We evaluated whether a delay in diagnostics and/or treatments was caused due to the absence of important information and what additional actions had to be taken to acquire these data (e.g., calling relatives). Finally, we assessed whether and how diagnostics and/or treatments were altered due to missing background information. The ER assessment was designed as a multiple-choice questionnaire to reduce the time necessary to complete it. Due to the patient anonymity, we could not gain further information on the delays/therapeutic consequences that were reported in the questionnaires.

#### 2.2.2. Inpatient Survey (*n* = 149)

The inpatient survey was obtained from geriatric patients who were admitted to our neurological ward or recruited via Parkinson support groups using questionnaires (22 questions total) (Figure 1). A special focus was put on PD patients (*n* = 38). The survey contained multiple-choice questions, but patients were also asked to give qualitative answers. Demographic features and information on the living and legal care status of the participants were collected. Additionally, we recorded information on comorbidities, medication, and the existence of documents like advanced directives or health care proxies. We investigated the participants’ current strategy of handling medical information. We handed out an emergency box and gave the according instructions on the use of the device.

#### 2.2.3. Follow-Up after 6 Months (*n* = 124)

After 6 months, we followed up the same participants who had performed the inpatient survey, asking about their experience with the emergency box (17 questions total, mixed multiple-choice and qualitative answers). One point of concern was whether the participants had used the emergency box correctly and whether changes of the medication/diagnoses or of important documents had been included into the box. We also assessed whether the living situation or legal care situation of the participants had changed in the meantime (e.g., moving into a nursing home), or whether the medical care had to be increased at home. Furthermore, we asked whether admissions to the ER had occurred in the interval and whether the emergency box had brought a subjective benefit in those situations. Finally, we investigated whether the participants subjectively liked the handling of the emergency box, whether it increased their feeling of safety, and if they were willing to continue using it.

### 2.3. Emergency Box

**Figure 2:**
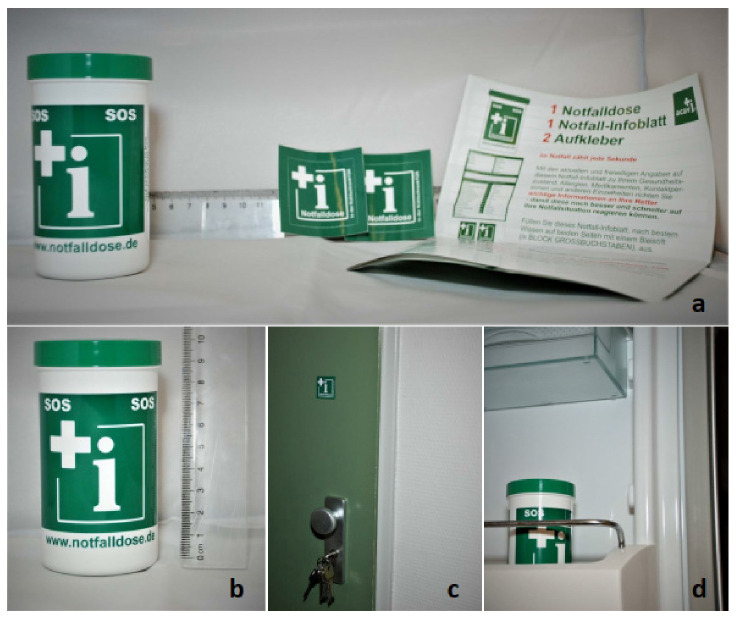
The emergency box and the instructions of its use. (**a**) shows the emergency box with its stickers and inlet; (**b**) shows the size of the emergency box. The stickers are supposed to be stuck on the front door (**c**) and the emergency box is supposed to be stored in the fridge door (**d**).

The emergency box is a small plastic device of white and green color that contains an inlet (Figure 2a,b) including necessary medical and personal information (Table 1). The inlet should contain personal data, allergies, important diagnoses, pre-existing disabilities, vaccinations, past operative procedures, medication (e.g., anticoagulants), phone numbers in case of emergency, and data on the general practitioner/nursing care service. The emergency box is supposed to be stored in the fridge door (Figure 2d). The stickers that come with the emergency box are stuck to the outside and inside of the front door and to the fridge door to alert the rescue services of its existence in the household (Figure 2c). In case of an emergency at home, the rescue services are supposed to bring the emergency box to the ER to provide the doctors with all important patient information upon arrival.

### 2.4. Statistical Analysis

All data are displayed as mean ± standard deviation and were calculated using Excel^®^ (Microsoft Cooperation, US, Redmond, DC, USA).

## 3. Results

### 3.1. ER Survey (n = 102)

All relevant information from the initial ER assessment is displayed in Table 2. In total, 53.9% of our patients in the ER survey were men; the mean age was 82.3 ± 5.2 years. In total, 27.4% of the geriatric patients came to the ER without a medication scheme. Only 4.9 % could remember their medication correctly while the others had problems recalling the appropriate medication name, times of intake, and/or dose. In 24.5% of all cases, the ER doctors had to call relatives or general practitioners to acquire important information, causing a delay in the acute care of the patients. Relevant delays in diagnostics or treatments in the ER and their reasons were reported in 11.8% (Table 2). Further specification of the reasons for the delay was not possible due to the anonymous multiple-choice assessment. In one case, administration of a contraindicated therapy occurred due to missing information, which could have resulted in a potentially life-threatening complication. Further problems in the ER were misjudgment due to lack of information in five cases (4.9%), relevant delay in treatment in four cases (3.9%), and additional unnecessary diagnostics in three cases (2.9%). In total, 55% of the emergency admittances occurred outside the regular working hours. Overall, the survey showed that there were complications and hindrances in the acute care of the patients due to the absence of important medical data in an emergency. 

### 3.2. Inpatient Survey (n = 149)

#### 3.2.1. Demographics, Comorbidities, and Medication

**Figure 3:**
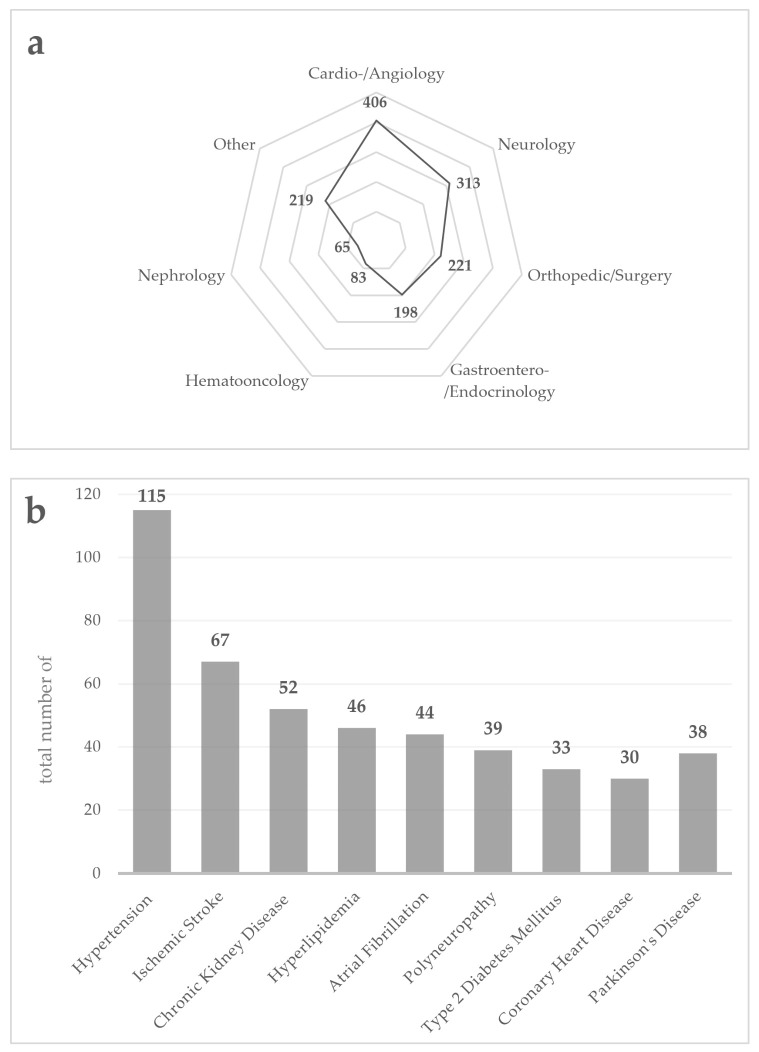
Comorbidities of the inpatient cohort (*n* = 149). (**a**) shows the number of major comorbidities in different medical disciplines as absolute numbers; (**b**) shows the number of the most common comorbidities as absolute numbers.

Participants of our inpatient cohort were 78.8 ± 5.4 years old. In total, 54.4% of the inpatients were men. The mean number of comorbidities was 10.1 ± 4.3. The most frequent comorbidities were hypertension, ischemic stroke, and chronic kidney disease (Figure 3). In PD patients (*n* = 38), the most frequent comorbidities were hypertension, chronic kidney disease, and polyneuropathy. The mean number of regularly taken drugs in our cohort was 7.7 ± 2.4. The most frequently used drugs were HMG-CoA-reductase-inhibitors, β-blockers, and AT 1 receptor antagonists. The most frequent drugs among the PD patients were Levodopa, β-blockers, and HMG-CoA-reductase-inhibitors.

#### 3.2.2. Inpatient Assessment

The inpatient assessment revealed that 76.5% of the participants claimed to have a medication list at home and 41.6% carried a medication list with them (e.g., in the wallet). In total, 34.2% failed to bring a medication scheme to the hospital admission. In total, 28.2% claimed to know their medication, doses, and time of intake, and 62.0% of those who had known allergies claimed to know them by heart. In total, 67.8% had a patient will, and only 28.7% of those brought the patient will to the hospital. Accordingly, health care proxies were owned by 64.4% of the participants, and only 21.9% brought them to the hospital admission.

The concept of the emergency box was familiar to 30.9% of our inpatient cohort. In total, 96.6% agreed to use the emergency box as instructed to take part in our study, and they were contacted after 6 months for the follow-up investigation.

### 3.3. Follow-Up after 6 Months

The follow-up investigation was completed by 124 of 149 (83.2%) of the participants. Five participants moved into a nursing home, and one of them was still able to complete the follow-up. In total, 23.4% had to adjust their medical care situation at home by hiring a nursing service or accelerating the frequency of nurse visits. Unfortunately, six participants died in the meantime. Three participants declined to take part in the follow-up, and 12 participants could not be reached by telephone.

The detailed results of the follow-up investigation are presented in Table 3. In general, about two thirds of the participants claimed to have used the emergency box according to the instructions. The idea behind the emergency box is for the rescue services to take the emergency box to the ER in case of an emergency at home without the help of the patient or his relatives. If the patient does not stick to the recommendations on the use of the emergency box, the concept can be jeopardized. Thus, it may be impossible for the rescue services to be aware of the existence of the emergency box and to find it. The emergency box is supposed to be stored in the fridge; whenever a patient stored it somewhere else, this was marked as “no” = incorrect use. The stickers are meant to be applied to the front and fridge door; whenever a patient did not apply them there or not at all, this was marked as “no” = incorrect use. Concerning the inlet, we asked whether patients filled it out correctly and if changes (e.g., of the medication) were included in the inlet as a correct update whenever necessary. Only if all of these points were met accurately, there was a chance, in case of an emergency at home, for the rescue services to find and bring the emergency box to the hospital. In total, 62.1% reported a change of their medication and 39.5% of those failed to update the emergency box accordingly. In total, 41.1% had added further documents to the emergency box. Most relevant problems regarding the use of the emergency box were the correct application of the stickers (not accomplished by 35.5%) and the update of the inlet (not accomplished by 39.5%). Overall, 83.1% were satisfied with the handling of the emergency box. 

Minor problems with the handling were losing the emergency box, dislike of the design (size of the box), and preference to remain in the current data management system (e.g., folder with health care data).

One focus of the follow-up study was admittances to the ER in the investigational period. In total, 29 of the patients (23.4%) were admitted at least once to the ER, and 20 of those were admitted from their home by rescue services. The others were either admitted from the office of their general practitioner or were brought to the ER by relatives. The emergency box was brought to the ER by the rescue services in only 4 of the 20 cases. All of these four patients reported that they benefited from the emergency box in the ER. They described the situation as less stressful and reported a time saving.

In total, 31.5% of all participants claimed that the possession of the emergency box increased their feeling of safety. Overall, 87.9% of patients were pleased with the concept of the emergency box and wanted to continue using it.

## 4. Discussion

Our hypothesis that there is a demand for a data management system for medical information of geriatric patients was confirmed, as almost one-third of the surveyed patients were admitted to the ER without medical data available. Acquiring the necessary information caused delays in the acute care of these patients. Even potentially dangerous complications were observed in our cohort. Additionally, patients reported a subjective benefit and an increased feeling of safety in possession of the emergency box.

The workflow in the ER is strongly disturbed if important information on the diagnoses, medication, and allergies are missing upon admission. This can result in reduced patient safety and false treatment decisions. We foremost observed a timely delay in the acute care due to missing medical data. The ER doctors had to make phone calls to acquire the information in almost one-fourth of the cases, which delayed the accurate care of the patients. We also observed potentially dangerous complications like mistakes in diagnostics and treatments due to a lack of information that clearly compromised patient safety in acute care. The absence of information about long-term medication can complicate various acute treatments, as the medication can be crucial to assess contraindications for treatment options.

There is increasing evidence that patients under polypharmacy are often overwhelmed by handling their medication and are not able to maintain an overview without help [9]. Especially if medication schemes were modified in an inpatient setting, the patients often did not know about their new medication in follow-up investigations [10,11]. Adding to this, general practitioners regularly change medication schemes shortly after discharge from the hospital [11]. Drug safety issues due to polypharmacy and the resulting hazard of drug interactions were reported frequently, especially for geriatric multimorbid patients [4,12,13]. A data management system like the emergency box has the potential to discover and solve drug interaction problems in acute and inpatient care.

In emergencies, it can be difficult for patients to remember important information as they are under a great amount of stress. It gets even more difficult if patients reach the ER unconsciously and cannot give any information. Language barriers are encountered often and can complicate taking the medical history [14]. It can be frustrating for both doctors and patients if information is desperately needed and the patient is not able to provide it. The emergency box has the potential to solve this dilemma.

The number of geriatric patients is rising in our industrial societies [15]. These patients often develop acute symptoms with the need for emergency admission [16,17]. PD patients are also likely to suffer from disease complications calling for acute diagnostics and treatments [18,19,20]. Geriatric and PD patients in our study had a mean number of 10.1 ± 4.3 comorbidities. Similar data were obtained in comparable studies before [13,21,22]. These results underline the high risk of these patients for recurrent and acute visits to hospitals [5]. Indeed, almost a quarter of our survey participants had to be admitted to the ER in the 6-month follow-up period. Therefore, the number of older people that could potentially benefit from an emergency box is high. If this concept is more established and the use in acute situations is successful more often, a high percentage of ER admissions could be avoided.

Our data also shows that better education and training of the rescue services is necessary to ensure the usage of the emergency box. The rescue services only brought the emergency box to the ER in 4 of 20 emergency admittances from the patients’ home. More education of general practitioners would be also helpful as our cohort was in need of support in handling the emergency box correctly and especially updating the contents.

Another important aspect touched by this study is the autonomy of geriatric patients who can be compromised in emergencies due to missing information. In total, 67.8% of our participants reported having an advanced directive. If this document is not available immediately upon ER admittance and the patient cannot comment on his will, the ER doctor has to decide on diagnostics and treatment according to the suspected will of the patient. This can lead to diagnostics or treatments that are not within the actual wishes of the patient [23]. Previous studies reported that a high amount of PD patients have advanced directives, so their use in the inpatient setting should be ensured [24,25]. The emergency box could be of help here, as the advanced directive is mentioned in the inlet as well.

Within the general context of the emergency box, it needs to be discussed that a comprehensive digital data management system is expected in the future and actually works in a few European countries, for example, in Denmark and Estonia [26]. In other countries, such as Germany, the electronic health card can theoretically be equipped with a medication plan and emergency data (allergies, pre-existing diseases, and an emergency contact) since August 2020 [27], but it has not been rolled out comprehensively so far. The implementation process of such digital systems develops slowly not only in Germany, mainly due to privacy and security requirements, the need for data protection, law amendments, and lack of political interests [28]. A digital concept making medical data accessible for doctors even in emergencies would make the emergency box obsolete. However, at the moment, there is no functioning concept foreseeable, so we argue that the emergency box is an easy and useful tool for the time until such a concept is developed and implemented comprehensively.

There are some limitations to our study. The initial ER survey was performed by a subset of neurological doctors and not by the total hospital team. Thus, selection bias cannot be excluded. A systematic review of all patients coming to the ER in a predefined period would be the next investigational step. Due to the lack of a control group and the exploratory nature of the study, statistical comparison was not possible. Furthermore, the monocentric design for this pilot project should be expanded to a multicentric and multidisciplinary design, since the emergency box does not only seem applicable for neurological patients.

## 5. Conclusions

Our results show that the emergency box might be useful in the emergency setting as long as no fully comprehensive digital data management exists. A clear advantage of the device is its price of less than 3€, its easy application, and that it increases the feeling of safety of geriatric patients. The workflow of the acute care in the ER can be fastened and facilitated for patients and doctors using the emergency box. Further investigations are necessary to determine whether the use of the emergency box can increase patient/drug safety and autonomy in the emergency setting. Data of future studies using the emergency box might also help in the development of digital drug safety solutions.

## Figures and Tables

**Table 1:** Information to be included in the inlet of the emergency box.

	Information
1.	Address and personal data
2.	Allergies
3.	Important diagnoses
4.	Vaccinations
5.	Preexisting disabilities (e.g., blindness, deafness)
6.	Important past operative procedures
7.	Medication
8.	Information on the general practitioner
9.	Information about the patient will and where it can be found
10.	Information on an organ donation statement and where it can be found
11.	Information about a nursing care service
12.	Photograph of the patient
13.	Emergency contact data
14.	Information on the existence of a pet and contact data of an emergency pet caregiver

**Table 2:** ER survey (*n* = 102). Note multiple answers for further explanation of the delay in the ER were possible.

	*n*	%
Medication scheme was brought to the ER		
Yes	74	72.6
No	28	27.4
Medication was remembered correctly		
Yes	5	4.9
No	97	95.1
Calling relatives/general practitioners was necessary		
Yes	25	24.5
No	76	74.5
Not specified	1	0.98
Delay of diagnostics/treatment		
Yes	12	11.8
No	90	88.2
Further explanation of the delay		
Misjudgment due to lack of information	5	35.7
Additional unnecessary diagnostics	4	28.6
Relevant delay in treatment	3	21.4
Contraindicated therapy	1	7.1
Relevant delay in general	1	7.1

**Table 3:** Use of the emergency box (follow-up investigation, *n* = 124).

	*n*	%
Admission to the ER in the follow-up period		
Yes	29	23.4
No	95	76.6
Transportation to the ER		
From home, through rescue services	20	69
From home, brought by relatives	4	13.8
Admission from a medical practice	5	17.2
The emergency box was used by rescue services		
Yes	4	20
No	16	80
Facilitated admission due to the emergency box		
Yes	4	100
No	0	0
Correct application at the patients’ home		
Storage in the fridge		
Yes	88	71
No	36	29
Stickers were used correctly		
Yes	79	63.7
No	44	35.5
Not specified	1	0.8
Inlet was filled out correctly		
Yes	109	87.9
Partially	8	6.5
No	3	2.4
Not specified	4	3.2
Inlet was updated		
Yes	29	23.4
Not necessary	43	34.7
No	49	39.5
Not specified	3	2.4
Further documents, e.g., medication list, were added		
Yes	51	41.1
No	70	56.5
Not specified	3	2.4
Increased feeling of safety		
Yes	39	31.5
No	77	62.1
Not specified	8	6.5
Further use in the future		
Yes	109	87.9
No	10	8.1
Perhaps	2	1.6
Not specified	3	2.4
Satisfied with the handling		
Yes	103	83.1
No	6	4.8
Not specified	15	12.1

## Data Availability

Data are available on reasonable demand to the corresponding author.
